# Supplementary material for: ProteinShader: illustrative rendering of macromolecules
Source: BMC Struct Biol. 2009 Mar 30;9:19. doi: 10.1186/1472-6807-9-19 (PMC2672931; doi:10.1186/1472-6807-9-19)
Supplement: Additional file 1 — ProteinShader program without source code. This compressed file contains the complete ProteinShader program including associated libraries, but no source code. A README.txt file gives an overview of the ProteinShader distribution, and the index.html file in the help subdirectory has directions on getting started with the program as well as a set of tutorials. [file 1472-6807-9-19-S1.zip › ProteinShader-beta-0_9_4-binary/help/api/org/proteinshader/gui/listeners/menubar/ToolsMenuListenerFactory.html]

ToolsMenuListenerFactory (ProteinShader API)


|  |  |  |
| --- | --- | --- |
| **Overview** \| **Package** \| **Class** \| **Use** \| **Tree** \| **Deprecated** \| **Index** \| **Help** | |  |
| **PREV CLASS**   **NEXT CLASS** | **FRAMES**    **NO FRAMES**     **All Classes** |  |
| SUMMARY: NESTED \| FIELD \| CONSTR \| METHOD | DETAIL: FIELD \| CONSTR \| METHOD |  |


---


## org.proteinshader.gui.listeners.menubar Class ToolsMenuListenerFactory

```
java.lang.Object
  org.proteinshader.gui.listeners.menubar.ToolsMenuListenerFactory
```

---

``` public class ToolsMenuListenerFactory extends Object ```

Creates all event listeners needed by the ToolsMenu of the
MainMenuBar.

---

**Constructor Summary**

`ToolsMenuListenerFactory(Mediator mediator)`
  
          Constructs a ToolsMenuListenerFactory.


| **Method Summary** | |
| --- | --- |
| `ItemListener` | `createControlPanelCheckBoxListener()`             Creates an item listener for the "Control Panel" check box menu item. |

**Methods inherited from class java.lang.Object**

`clone, equals, finalize, getClass, hashCode, notify, notifyAll, toString, wait, wait, wait`

**Constructor Detail**

### ToolsMenuListenerFactory

```
public ToolsMenuListenerFactory(Mediator mediator)
```

:   Constructs a ToolsMenuListenerFactory.

    **Parameters:**: `mediator` - the centralized Mediator object that listeners call on to accomplish their task.


**Method Detail**

### createControlPanelCheckBoxListener

```
public ItemListener createControlPanelCheckBoxListener()
```

:   Creates an item listener for the "Control Panel" check box menu
    item. When the item listener is called, it will open the control
    panel frame if the check box is selected or hide it if the check
    box is deselected.

    :   **Returns:**: The item listener.


---


|  |  |  |
| --- | --- | --- |
| **Overview** \| **Package** \| **Class** \| **Use** \| **Tree** \| **Deprecated** \| **Index** \| **Help** | |  |
| **PREV CLASS**   **NEXT CLASS** | **FRAMES**    **NO FRAMES**     **All Classes** |  |
| SUMMARY: NESTED \| FIELD \| CONSTR \| METHOD | DETAIL: FIELD \| CONSTR \| METHOD |  |


---

# *Copyright © 2007-2008*
